# Estimation of the relationship between meteorological factors and measles using spatiotemporal Bayesian model in Shandong Province, China

**DOI:** 10.1186/s12889-023-16350-y

**Published:** 2023-07-25

**Authors:** Yan Jia, Qing Xu, Yuchen Zhu, Chunyu Li, Chang Qi, Kaili She, Tingxuan Liu, Ying Zhang, Xiujun Li

**Affiliations:** 1 Department of Biostatistics, School of Public Health, Cheeloo College of Medicine, Shandong University, Jinan, 250012 China; 2 Institute of Immunization and Preventive Management, Shandong Center for Disease Control and Prevention, Jinan, 250014 China; 3 Faculty of Medicine and Health, School of Public Health, University of Sydney, Camperdown, NSW 2006 Australia

**Keywords:** Measles, Meteorological factors, Spatiotemporal Bayesian model, Regional relative risks

## Abstract

**Background:**

Measles-containing vaccine (MCV) has been effective in controlling the spread of measles. Some countries have declared measles elimination. But recently years, the number of cases worldwide has increased, posing a challenge to the global goal of measles eradication. This study estimated the relationship between meteorological factors and measles using spatiotemporal Bayesian model, aiming to provide scientific evidence for public health policy to eliminate measles.

**Methods:**

Descriptive statistical analysis was performed on monthly data of measles and meteorological variables in 136 counties of Shandong Province from 2009 to 2017. Spatiotemporal Bayesian model was used to estimate the effects of meteorological factors on measles, and to evaluate measles risk areas at county level. Case population was divided into multiple subgroups according to gender, age and occupation. The effects of meteorological factors on measles in subgroups were compared.

**Results:**

Specific meteorological conditions increased the risk of measles, including lower relative humidity, temperature, and atmospheric pressure; higher wind velocity, sunshine duration, and diurnal temperature variation. Taking lowest value (Q1) as reference, RR (95%CI) for higher temperatures (Q2–Q4) were 0.79 (0.69–0.91), 0.54 (0.44–0.65), and 0.48 (0.38–0.61), respectively; RR (95%CI) for higher relative humidity (Q2–Q4) were 0.76 (0.66–0.88), 0.56 (0.47–0.67), and 0.49 (0.38–0.63), respectively; RR (95%CI) for higher wind velocity (Q2–Q4) were 1.43 (1.25–1.64), 1.85 (1.57–2.18), 2.00 (1.59–2.52), respectively. 22 medium-to-high risk counties were identified, mainly in northwestern, southwestern and central Shandong Province. The trend was basically same in the effects of meteorological factors on measles in subgroups, but the magnitude of the effects was different.

**Conclusions:**

Meteorological factors have an important impact on measles. It is crucial to integrate these factors into public health policies for measles prevention and control in China.

**Supplementary Information:**

The online version contains supplementary material available at 10.1186/s12889-023-16350-y.

## Introduction

Measles is a prevalent acute respiratory infectious disease in children caused by measles virus, which is transmitted through respiratory droplets, as well as direct and indirect contact between individuals [1]. The main clinical symptoms of measles infection include fever, rash, cough, and the presence of oral mucosal spots. In some cases, complications such as pneumonia, diarrhea, and keratoconjunctivitis may arise [2]. Measles is a significant infectious disease that can lead to fatal outcomes in children. Unfortunately, there is currently no specific treatment available for measles. Strengthening disease surveillance and enhancing vaccination coverage are considered highly cost-effective public health interventions.

While worldwide measles immunization campaigns have achieved remarkable results, the incidence of measles remains high in certain countries and regions with less developed economies and limited access to healthcare, such as Africa and Southeast Asia [3]. The accumulation of a susceptible population and the high contagiousness of measles pose challenges in its elimination. Since 2016, measles has rebounded in some countries that include developed countries. The global number of measles cases had increased from 132,490 in 2016 to 869,770 in 2019 [3]. Notably, China has been among the top three reporting countries for measles cases between 2010 and 2020 [4].

In China, since 1986, a routine immunization program for two doses of measles vaccine has been implemented [5], with the government bearing the cost. In 2005, China's national recommended age for two doses of measles-containing vaccine (MCV) has been adjusted to 8 months and 18–24 months [6]. Immunization coverage with MCV second-dose (MCV2) by the nationally recommended age has consistently been over 95% since 2008 [7]. China has set a goal of eliminating measles by 2012, but experienced a resurgence after that, with the highest incidence occurring in 2014 at 3.9 cases per 100,000 (52,628 cases) [8]. In recent years, the age group with the highest incidence has shifted from children under 5 years old to infants and young-adults [8, 9]. Shandong Province in China, with its high population density and contact rate, has faced challenges in eliminating measles and ranked first among all provinces in 2016 for the number of measles cases, with an incidence rate of 4.5 per 100,000 [10]. It is crucial for relevant health departments to maintain vigilance in prevention and control of measles.

Previous studies on infectious disease dynamics analysis have mainly focused on the impact of demographic factors on the incidence of measles, such as birth rate, age structure, vaccination coverage, and social contact patterns [11–13]. It is noted that the transmission of measles is also affected by other factors, including the survival time and activity of measles virus under various environmental conditions, which are related to meteorological factors [14]. Existing evidence suggests that meteorological factors, such as temperature and humidity, may have a nonlinear effect on measles, but findings have not been consistent [15, 16]. Meteorological conditions vary across regions, and the measles virus is influenced by multiple weather factors, increasing the complexity of the study and possibly contributing to inconsistent results. Studies in Niger has found a negative correlation between rainfall and measles cycle [17, 18]. Measles transmission may also be related to wind velocity, as dust storms have been associated with the transport of infectious diseases. At present, the direct quantitative research on the impact of meteorological factors on measles is very limited. It is beneficial to explore the impact of meteorological factors on measles to provide reference for future research. Meanwhile, researches have showed significant spatial heterogeneity and clustering of measles distribution [20, 21]. Therefore, the spatial and temporal effects should be comprehensively considered when studying the impact of meteorological factors on measles.

This study aims to assess the impact of meteorological factors on measles and identify high-risk regions in Shandong Province. According to meteorological change, the measles prevention and control should be strengthened among key population in key areas, to provided scientific evidence for relevant departments.

## Materials and methods

### Study area

Shandong Province, located in the eastern coast of China, experiences a warm temperate monsoon climate. It covers a land area of 155,800 square kilometers and had a total population of 97,894,300 at the end of 2014 (http://www.shandong.gov.cn/) (accessed on 28 October 2021). Shandong Province is administratively divided into 137 counties (districts). A total of 136 counties (districts) were selected as the study objects, excluding Changdao County due to its lack of neighboring counties within the jurisdiction of Shandong Province.

### Data collection

Data on measles cases from 2009 to 2017 were obtained from China Information System for Disease Control and Prevention (CISDCP). Case data included information of age, sex, occupation, time of onset, and residential county. Cases without valid residential county data were excluded from the analysis.

Meteorological data were extracted from National Meteorological Information Center (http://data.cma.cn/) (accessed on 28 October 2021). Variables include average daily precipitation (mm), average daily atmospheric pressure (hPa), average daily wind velocity (m/s), average daily relative humidity (%), sunshine duration (h), average daily temperature (℃) and daily diurnal temperature variation (℃). Diurnal temperature variation was calculated as the difference between the daily maximum temperature and daily minimum temperature. Find the monthly mean of daily meteorological data to obtain monthly data. We obtained the same period data from all meteorological stations in Shandong Province. Kriging interpolation method was employed to obtain meteorological data of all counties (districts) [22].

### Data analysis

Since the impact of meteorological variables on the incidence of measles is nonlinear, when used as continuous variables, nonlinear modeling will increase the model complexity and decrease interpretability. Additionally, when the sample size is small, nonlinear modeling may lead to overfitting. To address these issues, meteorological variables were converted into categorical variables using the quartile method, which were Q1–Q4, representing the lowest (Q1), medium–low (Q2), medium–high (Q3) and highest (Q4) levels of variable values respectively. Spearman correlation analysis was carried out to examine the correlation between covariates. Spearman correlation coefficients (*r*_*s*_) between covariates greater than 0.8 were considered highly correlated, indicating the possibility of multicollinearity. Covariates with large correlation coefficients would be excluded to avoid the occurrence of multicollinearity and improve the accuracy of model estimation. Spatiotemporal Bayesian model was used to estimate the effects of meteorological factors on measles, and evaluate measles risk areas at county level. The relative risk (RR) of measles virus infection in Q2–Q4 all taken Q1 as a reference to reflect the impact of meteorological factors at different levels on measles. The 95% credibility interval (CI) of RR was used to determine statistical significance. Total case population was divided into multiple subgroups according to genders, ages and occupations. Subgroups were modeled separately. The effects of meteorological factors on measles among these subgroups were compared.

Spatiotemporal Bayesian model is suitable for small areas spatial analysis. It incorporates prior information and utilizes the integrated nested Laplace approximations (INLA) algorithm to estimate unknown parameters. This model takes spatial proximity information and temporal correlation into account, enabling the quantification of specific regional disease risks and exploration of related influencing factors. It has been widely used in infectious disease modeling [23, 24], like the analysis of German measles [25].

Due to the excessive number of zero values and potential over-dispersion in the monthly number of cases at the county scale, we adopted the zero-inflated negative binomial spatiotemporal Bayesian model. Spatiotemporal Bayesian model approximates the posterior marginal of the model effects by the INLA algorithm [26, 27]. The covariate effects were presented by relative risk (RR), $\mathrm{RR}=\mathrm{exp}(\upbeta )$, and the risks in different areas were visualized through the maps of specific district relative risk and posterior probability [28, 29]. Medium–high-risk areas were defined as counties (districts) with a posterior mean of specific districts relative risk (RR) > 2 and a posterior probability ≥ 0.7. High-risk areas were defined as the counties (districts) with posterior mean of relative risk (RR) > 3, and posterior probability ≥ 0.7 [25]. Fitness of the models were assessed using the deviance information criterion (DIC), Watanabe–Akaike information criterion (WAIC), conditional predictive ordinate (CPO), marginal log-likelihood (MLIK) and mean square error (MSE).

The zero-inflated negative binomial spatiotemporal Bayesian model is formulated as follows. For different distributions (Poisson distribution, negative binomial distribution, etc.), the zero-inflated model has different definitions. Assuming the presence of structural and sampling zeros in the data, its probability function is expresses as [30]

$$\mathrm{p}\left({y}_{it}|{\pi }_{0}\right)= {\pi }_{0}I\left({y}_{it}=0\right)+(1-{\pi }_{0})\times \mathrm{g}({y}_{it})$$

When ${y}_{it}$ obeys a negative binomial (NB) distribution, there is

$$\mathrm{g}\left({y}_{it}\right)=\frac{\Gamma \left({y}_{it}+{n}_{it}\right)}{\Gamma \left({n}_{it}\right)\Gamma \left({y}_{it}+1\right)}{{{p}_{it}}^{{n}_{it}}(1-{p}_{it})}^{{y}_{it}}$$

${y}_{it}(i=\mathrm{1,2},\dots ,136;t=\mathrm{1,2},\dots ,9)$ is the number of observed cases in area *i* time *t*. $I\left({y}_{it}=0\right)$ is indicator variable, ${\pi }_{0}$ is probability of observing zero. The zero-inflated parameter ${\pi }_{0}$ is represented as ${\pi }_{0}=\frac{\mathrm{exp}({\theta }_{2}) }{1+\mathrm{exp}({\theta }_{2})}$. ${p}_{it}$ is probability of success in each trial. ${n}_{it}$ is number of successful trials, which is dispersion parameter and is represented as ${n}_{it} =\mathrm{exp}({\theta }_{1})$. ${\pi }_{0}$ and ${n}_{it}$ are hyperparameters. ${\theta }_{2}$ is the internal representation of ${\pi }_{0}$; ${\theta }_{1}$ is the internal representation of ${n}_{it}$.

Spatiotemporal Bayesian models were constructed to analyze the relationship between meteorological factors and measles. The model formula is as follows:

$$\mathrm{log}\left({\eta }_{it}\right)= {b}_{0}+{u}_{i}+{v}_{i}+{\gamma }_{t}+{\phi }_{t}+{\sum }_{m=1}^{M}{\beta }_{m}{x}_{m}+\mathrm{log}({E}_{it})$$

${\eta }_{it}$ is the expected number of cases in area *i* time *t*, ${\beta }_{1},\dots ,{\beta }_{M}$ are covariate parameters. ${u}_{i}$ is the area-specific spatially structured random effect, modeled using intrinsic conditional autoregressive (iCAR), ${v}_{i}$ is the area-specific spatially unstructured random effect, that is, exchangeable (independent and identically distributed, iid). The combination of ${u}_{i}$ and ${v}_{i}$ is Besag–York–Molliè (BYM) model [31, 32]. This study used BYM model to represent the spatially structured and unstructured random effects. ${\gamma }_{t}$ is the structured temporal random effect, modeled dynamically using a random walk of order 2 (rw2) to reflect the nonparametric dynamic trend of disease. ${\phi }_{t}$ is the unstructured temporal random effect, modeled using iid. $\mathrm{log}({E}_{it})$ is the offset, here is annual population of each county.

The prior information of parameters in spatiotemporal Bayesian model was in accordance with the default settings in “INAL” package of R, and detailed information was provided in Table 1. The statistical analysis was conducted using R (version 4.0.3), and the spatiotemporal Bayesian modeling analysis was performed using the “INLA” package.

**Table 1:** Specification of prior information

Parameters	Prior distribution	Initial value
Fixed effects	β ~ Gaussian (0, 10^–3^)	
Random effects		
Spatial (BYM)^a^	τ ~ Gamma (1, 5^a^ 10^–4^)	4
Temporal (iid)	τ ~ Gamma (1, 5^a^ 10^–5^)	4
Temporal (rw2)	τ ~ Gamma (1, 5^a^ 10^–5^)	4
Over-dispersion	θ1 ~ pc.mgamma (7)^b^	2.30258509299405
Zero-inflated	θ2 ~ Gaussian (-1, 0.2)	-1

^a^Two spatial parameters, including structured and unstructured spatial terms

^b^Penalised complexity prior (pc.mgamma prior)

## Results

Table 2 summarized the characteristics of cases of measles. From 2009 to 2017, a total of 15,647 cases of measles were reported in Shandong Province, with large variation in the number of cases across different years. The average male to female cases ratio was 1.32. The age groups < 5 and 20–39 accounted for approximately 80% of the cases, of which < 5 age group accounted for about 50%. Among different occupations, scattered children (Children who kept at home without going to a nursery, kindergarten, etc.) and farmers accounted for 72.6% of cases, of which scattered children accounted for 47.9%. Students accounted for 7.3% of cases. The proportion of each group varied over the nine-year period but did not exhibit significant changes.

**Table 2:** Epidemiological characteristics of measles in Shandong Province, 2009–2017

	2009	2010	2011	2012	2013	2014	2015	2016	2017	Total
Cases	2185	1759	484	21	1476	2245	2969	4404	104	15,647
Gender ratio (M/F)	1.53	1.56	1.56	2.50	1.47	1.2	1.24	1.19	1.67	1.32
Age (years) (%)										
< 5	48.6	57.0	53.3	66.7	61.9	45.4	50.9	40.4	60.6	48.7
5–19	12.5	7.2	6.2	4.8	4.0	5.7	4.9	7.3	8.7	7.0
20–39	34.1	29.6	31.6	19.0	25.7	35.3	28.4	34.6	17.3	31.8
≥ 40	4.8	6.2	8.9	9.5	8.4	13.6	15.8	17.7	13.4	12.5
Occupation (%)										
Scattered children	46.2	57.2	52.9	61.9	60.8	44.6	50.5	39.9	55.8	47.9
Students	13.1	6.4	7.4	14.3	4.9	6.3	5.1	7.5	14.4	7.3
Farmers	20.5	20.5	18.6	9.5	19.4	27.1	22.5	31.6	12.5	24.7
Workers	6.4	5.5	8.5	4.8	3.9	6.0	6.3	5.1	1.9	5.7
Housekeeping	2.8	3.1	3.9	0.0	4.4	5.9	5.9	5.9	4.8	4.9
Others	11.0	7.3	8.7	9.5	6.6	10.1	9.7	10.0	10.6	9.5

*M* Male, *F* Female. Students: including kindergarten children. Housekeeping: including unemployed

**Fig. 1:**
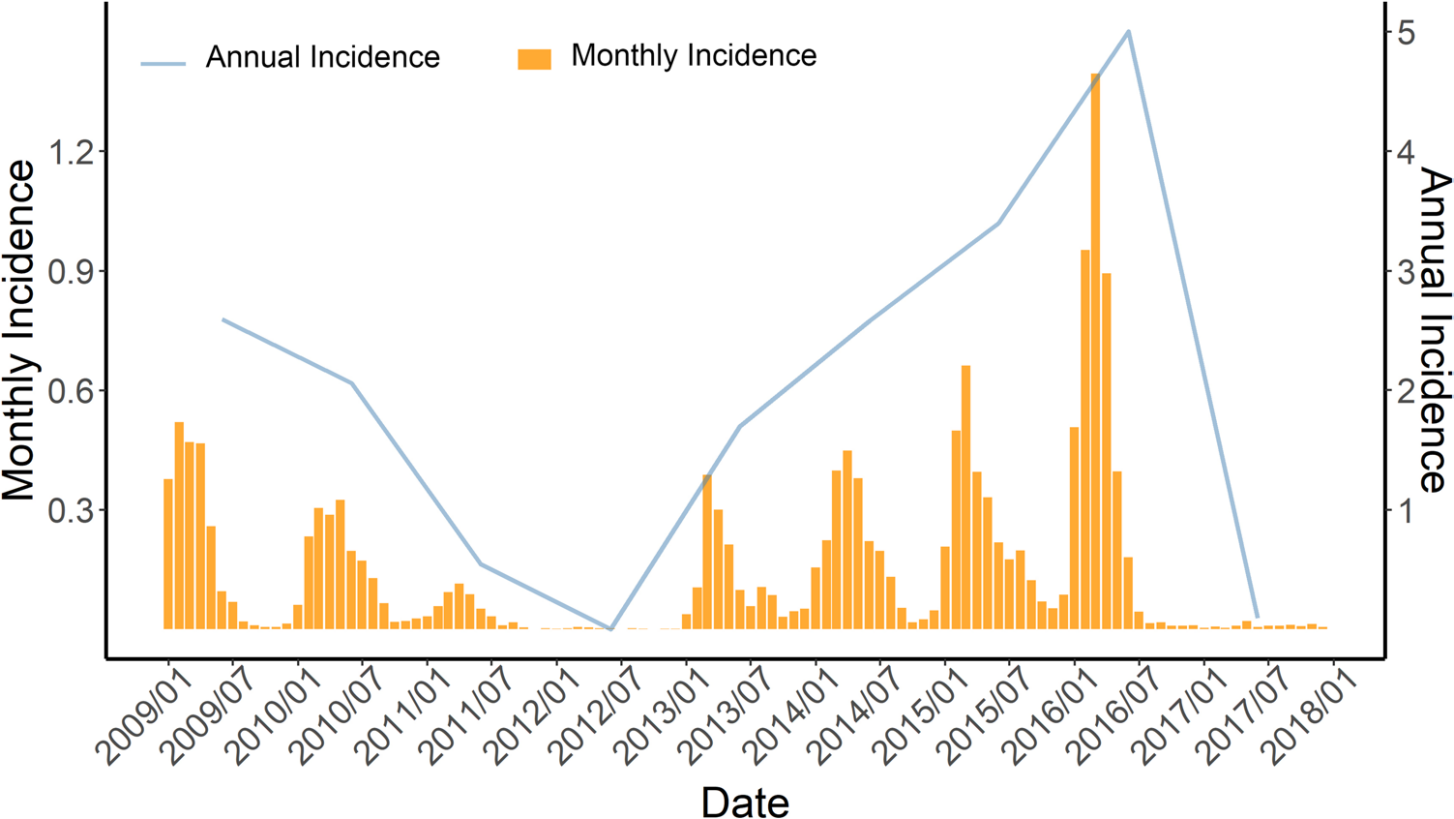
Incidence of measles per 100,000 in Shandong Province, 2009–2017

**Fig. 2:**
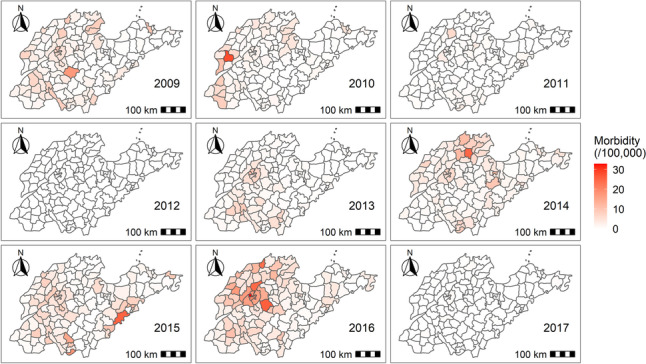
Maps of measles incidence at county (district) scale in Shandong Province, 2009–2017

The incidence of measles in Shandong Province had obvious seasonality, with high incidence occurring between January and June each year, peaking in March and April. The incidence of measles showed a downward trend in 2009–2012 but an upward trend in 2013–2016 (Fig. 1). From the perspective of geographical distribution, the incidence of measles was concentrated in western and middle Shandong, and in 2015, included individual counties along the eastern coast (Fig. 2).

Statistical description of the meteorological variables was shown in Supplementary Table S1. From 2009 to 2017, monthly average precipitation was 1.81 mm (range: -0.16–17.36 mm), average atmospheric pressure was 1000.40 hPa (range: 886.99–1037.23 hPa), average wind velocity was 2.64 m/s (range: 0.90–8.66 m/s), average relative humidity was 65.70% (range: 30.70–98.11%), average sunshine duration was 6.33 h (range: 1.13–10.94 h), average temperature was 13.32 ℃ (range: -9.27–28.92 ℃), average diurnal temperature variation was 9.12 ℃ (range: 3.20–14.08 ℃). The correlation analysis results were shown in Supplementary Table S2, indicating that all correlation coefficients between covariates were below 0.8, indicating no multicollinearity. Therefore, all variables were included in the model.

**Fig. 3:**
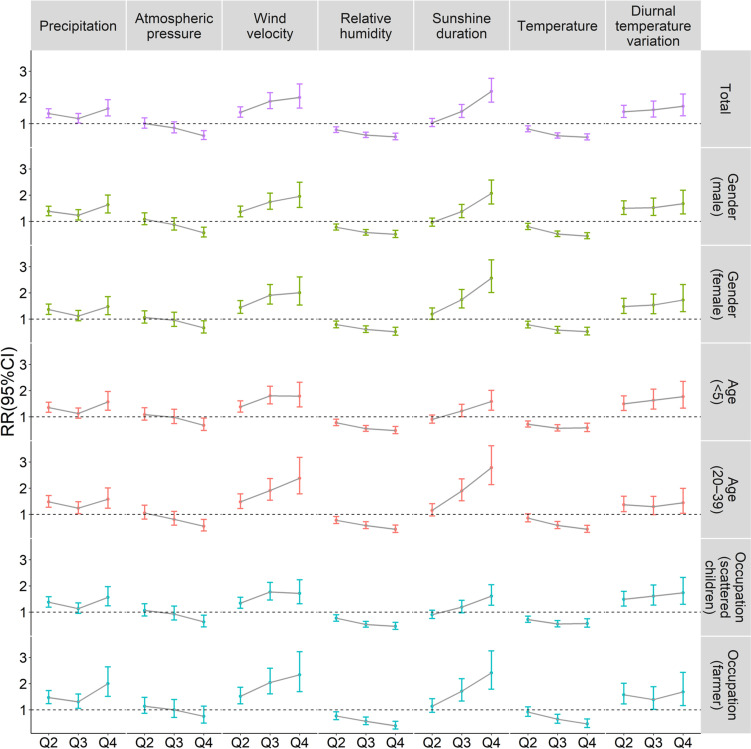
Associations between meteorological factors and measles incidence for different subgroups in Shandong Province, 2009–2017 (RR (95%CI); RR of meteorological factors takes the Lowest value of variable (Q1) as a reference)

A total of 7 spatiotemporal Bayesian models were established for total case population and various subgroups. Subgroups included cases by gender, high-risk age groups (< 5 and 20–39) and high-risk occupations (scattered children and farmers). The modeling results were shown in Fig. 3, and estimated relative risks were detailed in Supplementary Table S3. Estimated hyperparameters and measures of model fit were presented in Supplementary Table S4. The total model suggested that wind velocity, sunshine duration, and diurnal temperature variation were positively associated with measles, that is, the higher values of these variables corresponded to a higher district-level risk of measles virus infection. Wind velocity and sunshine duration exhibited significant influence on measles, while the effect of diurnal temperature variation was relatively small.

Atmospheric pressure, relative humidity, temperature were inversely associated with measles, that is, the higher of these variables corresponded to a lower district-level risk of measles virus infection. Taking the lowest level (Q1) of atmospheric pressure as reference, only the highest level (Q4) had a statistically significant impact on measles. The effects of relative humidity and temperature on measles were similar and more significant. Taking the lowest level (Q1) as reference, the RR (95%CI) for higher temperatures (Q2–Q4) were 0.79 (0.69–0.91), 0.54 (0.44–0.65), and 0.48 (0.38–0.61), respectively; the RR (95%CI) for higher relative humidity (Q2–Q4) were 0.76 (0.66–0.88), 0.56 (0.47–0.67), and 0.49 (0.38–0.63), respectively.

Taking the lowest level (Q1) as reference, a reduced risk of measles virus infection was found from the medium–low (Q2) to medium–high precipitation (Q3), but there was an increased risk from medium–high (Q3) to highest (Q4) precipitation level, showing a "V"-shaped correlation, with RR (95%CI) of 1.39 (1.23–1.57), 1.19 (1.03–1.39), and 1.57 (1.29–1.91), respectively.

The effects of meteorological factors on measles were generally consistent across different subgroups, although the magnitude of the effects varied. The female subgroup was more susceptible to the impact of sunshine duration and wind velocity compared to male subgroup. At the same level of sunshine duration and wind velocity, females had a higher risk of measles virus infection than males. Compared with other subgroups, the 20–39 age subgroup was least affected by diurnal temperature variation, but most affected by highest wind velocity (Q4) (RR: 1.66, 95%CI: 1.30–2.13). Sunshine duration exerted a lower impact on < 5 age and scattered children subgroup than other subgroups. Meanwhile, there was a reduced risk of measles virus infection for < 5 age and scattered children subgroup from medium–high (Q3) to highest (Q4) wind velocity, showing an inverted "V"-shaped correlation. The risk of measles virus infection in farmer subgroup was more susceptible to the impact of the highest (Q4) precipitation than other subgroups, while there was no statistical significance influence of different atmospheric pressure levels on farmers.

**Fig. 4:**
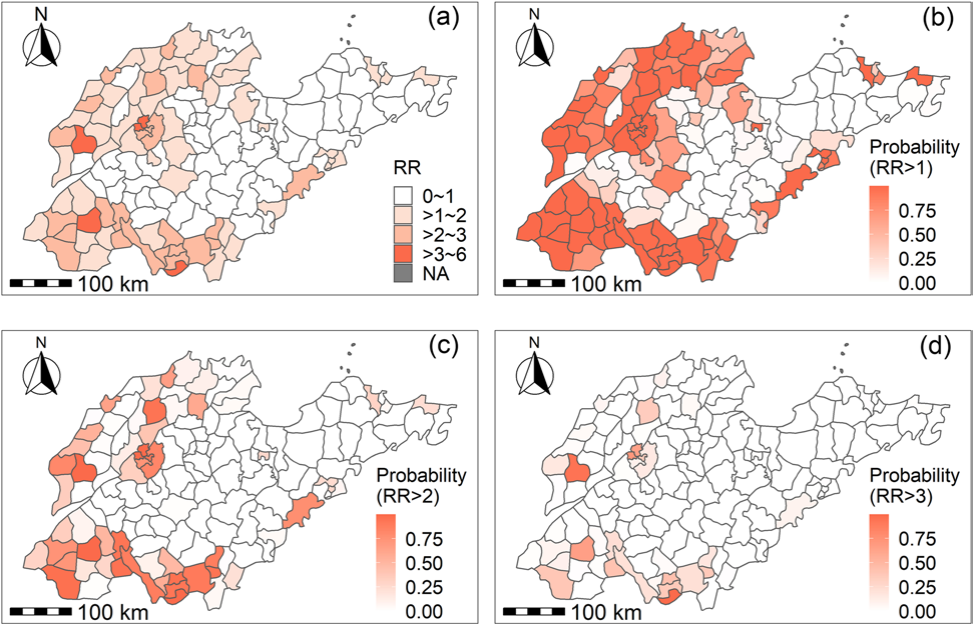
The maps of posterior mean of specific districts relative risks (RRs) and posterior probability in Shandong Province, 2009–2017

This study estimated specific districts relative risk of measles virus infection based on total model. The maps depicting the posterior mean of specific districts relative risk and posterior probability obtained by modeling were shown in Fig. 4. The study identified 22 measles medium–high-risk areas, as shown in Fig. 4c. The RR and posterior probability values of medium–high-risk counties were shown in Supplementary Table S5. The medium–high-risk counties (districts) of measles were mainly concentrated in northwestern, southwestern and central regions of Shandong Province. The cities with a concentration of medium–high-risk counties include Jinan [6], Heze [4], Zaozhuang [3], Jining [3], Linyi [3], Liaocheng [2], and Qingdao [1]. Amongthese counties, there were 3 measles high-risk areas, as shown in Fig. 4d: Taierzhuang District in Zaozhuang City, Dongchangfu District in Liaocheng City, and Huaiyin District in Jinan City. The posterior time trend for measles can be seen in Supplementary Figure S1. Model fitting value maps of measles cases in counties (districts) in Shandong Province from 2009 to 2017 were shown in Supplementary Figure S2.

## Discussion

This study has described the epidemiological characteristics of measles in Shandong Province and explored the impact of meteorological factors on measles. Regarding the epidemiological characteristics of measles, the decrease in measles cases in 2012 and 2017 might be related to the goal of eliminating measles by 2012 and the continuous implementation of the MMR vaccination policy. By establishing a spatiotemporal Bayesian model, this study has identified 22 medium–high-risk counties (districts) primarily located in northwestern, southwestern and central Shandong Province. The cities with concentrated medium–high-risk counties were Jinan, Heze, Zaozhuang, Jining, Linyi, Liaocheng, and Qingdao. Additionally, the effect of meteorological factors on measles has estimated, and compared in subgroups in this research.

The analysis revealed that increased wind velocity, sunshine duration, and diurnal temperature variation correlate with an elevated risk of measles. Similar conclusions have been drawn by previous studies [15, 33]. Research showed that the long-distance transportation of air pollutants, such as sandstorms caused by high winds, contributes to higher local measles virus aerosol level [34, 35]. Monthly average sunshine duration generally exceeded 5 h and peaked in April–June in Shandong Province [36]. A study conducted in a Southern Chinese city shown that the incidence of measles have a positive relationship with the sunshine duration exceeding 5 h [16]. The positive effect of sunshine duration on measles might be related to the promotion of outdoor activities and crowd movement under favorable meteorological conditions. Moreover, abrupt temperature changes can increase the risk of respiratory and cardiovascular diseases, induce inflammation in allergic rhinitis, affect the nasal mucosa [37, 38], which impact the resistance of respiratory system to the measles virus.

Higher atmospheric pressure, relative humidity, and temperature levels appeared to reduce the risk of measles virus infection and have a protective effect [16, 39]. In addition to ecological studies, explanations could be found in the measles viral characteristics. Virology studies have found that high relative humidity will reduce the survival rate and survival time of measles virus. Under 25–29% relative humidity, the survival time of measles virus is more than a dozen times longer than that in 68–70% relative humidity, and at 15 °C, the survival of measles virus is slightly better than that at 20 °C [14].

The effect of precipitation on measles was slightly more complex, exhibiting a V-shaped correlation. Previous studies have highlighted the impact of airflow patterns and ventilation rates on spread of pathogens in crowded indoor environments such as hospitals, schools, and shopping malls [40, 41]. Precipitation could affect the spread of measles virus by altering indoor environmental sanitation, ventilation and the deposition rate of pathogen containing aerosols [42]. Therefore, the relationship between precipitation and measles was influenced by various factors, such as regional differences and the combined effects of meteorological factors, which may explain the divergence from some previous studies [15, 16, 39]. It might also be related to the consideration of spatiotemporal effects in this study, which contains the inherent characteristics of time and region and weakens the influence of confounding factors.

Measles incidence demonstrated regional variation from year to year, potentially due to the accumulation and reduction of susceptible individuals within specific regions. The western region consistently exhibited higher measles incidence than the eastern region, consistent with the risk region obtained from the modeling results. The population with high-risk of measles included the age groups < 5 and 20–39, scattered children and farmers. This might be attributed to increased outdoor activities, gatherings [43], and a weaker resistance to measles virus in the absence of two doses of MCV vaccination [44, 45]. Compared to other subgroups, the risk of measles virus infection in adults aged 20–39 and farmers subgroups was more susceptible to meteorological factors, such as sunshine duration and wind velocity. Measles seroprevalence among young adults is low, and the effects of supplementary immunization activities (SIAs) are short-lived for adults [46]. Therefore, the protection provided by measles vaccine for adults is limited. Young adults aged 20–39 and farmers are more likely to spread the virus, particularly under favorable meteorological conditions, due to their frequent contact with others and high population mobility. The risk of measles virus infection was slightly more affected by Meteorological changes in females compared to males, although the mechanism of sex influence on measles remains inconclusive based on previous studies [47, 48].

This study considered spatiotemporal random effects when analyzing the relationship between meteorological factors and measles, enhancing the reliability of the results. However, there were some limitations. Limited by the lack of age-specific (or sex-specific) population data for each region, this study could only calculate the measles incidence and not the expected number of cases or standardized incidence ratio (SIR). Monthly data were used, but considering the incubation period of measles (generally 7–21 days), analyzing the lag effect may not be appropriate. Furthermore, this research focused primarily on the relationship between meteorological factors and measles, without considering social factors such as demographic economic factors and vaccination status. Therefore, it is important to consider the differences in these factors when extrapolating to other regions. Although there are few existing studies on influence of meteorological factors on measles, this paper helps to address this research gap to some extent. Additionally, the results of this study hold reference significance for other regions with a similar incidence pattern.

## Conclusion

Meteorological factors, especially temperature, relative humidity, wind velocity and sunshine duration, have an important impact on measles. It is important to integrate these factors into public health policies for measles prevention and control. For example, organizing measles education campaigns based on meteorological conditions.

## Supplementary Information

**Additional file 1.**

## Data Availability

Measles data supporting the results of this study were available from the Shandong Center for Disease Control and Prevention, but the availability of these data was limited, which was used under the license for the current study. If appropriate permission for the data is required, please contact the corresponding author.

## Abbreviations

MCV: Measles-containing vaccine
CISDCP: China Information System for Disease Control and Prevention
RR: Relative risk
CI: Credibility interval
INLA: Integrated nested Laplace approximations
iCAR: Intrinsic conditional autoregressive
BYM: Besag–York–Molliè
SIA: Supplementary immunization activities
