# Supplementary material for: Estimation of the relationship between meteorological factors and measles using spatiotemporal Bayesian model in Shandong Province, China
Source: BMC Public Health. 2023 Jul 25;23:1422. doi: 10.1186/s12889-023-16350-y (PMC10369697; doi:10.1186/s12889-023-16350-y)
Supplement: Supplementary file 1 — Additional file 1. [file 12889_2023_16350_MOESM1_ESM.docx]

**Supplementary material**

Estimation of the relationship between meteorological factors and measles using spatiotemporal Bayesian model in Shandong Province, China

Yan Jia ^1^, Qing Xu ^2^, Yuchen Zhu ^1^, Chunyu Li ^1^, Chang Qi ^1^, Kaili She ^1^, Tingxuan Liu ^1^, Ying Zhang ^3^ and Xiujun Li ^1,^*

^1^ Department of Biostatistics, School of Public Health, Cheeloo College of Medicine, Shandong University, Jinan 250012, China; jiayan8hui@163.com (Yan Jia); zhuyuchenl@163.com (Yuchen Zhu); lichunyu_biosta@163.com (Chunyu Li); chanzyq@163.com (Chang Qi); kailishe95@163.com (Kaili She); 201935820@mail.sdu.edu.cn (Tingxuan Liu);

^2^ Institute of Immunization and Preventive Management, Shandong Center for Disease Control and

Prevention, Jinan 250014, China; xqepi@163.com

^3^ Faculty of Medicine and Health, School of Public Health, University of Sydney, Camperdown, NSW 2006, Australia; ying.zhang@sydney.edu.au

* Correspondence: xjli@sdu.edu.cn; Tel.: +86-531-88382140

Table S1. Description of meteorological factors in Shandong Province, 2009–2017.

| Monthly meteorological data | *M* ± *SD* | *Min* | *P*_25_ | *P*_50_ | *P*_75_ | *Max* |
| --- | --- | --- | --- | --- | --- | --- |
| Precipitation (mm) | 1.81 ± 2.35 | -0.16 | 0.30 | 0.90 | 2.34 | 17.36 |
| Q1 | 0.10 ± 0.09 |  |  | 0.08 |  |  |
| Q2 | 0.57 ± 0.17 |  |  | 0.56 |  |  |
| Q3 | 1.49 ± 0.40 |  |  | 1.44 |  |  |
| Q4 | 5.05 ± 2.60 |  |  | 4.28 |  |  |
| Atmospheric pressure (hPa) | 1000.40 ± 25.01 | 886.99 | 994.91 | 1005.83 | 1016.49 | 1037.23 |
| Q1 | 967.62 ± 28.27 |  |  | 978.93 |  |  |
| Q2 | 1001.34 ± 2.87 |  |  | 1001.48 |  |  |
| Q3 | 1011.20 ± 3.16 |  |  | 1010.98 |  |  |
| Q4 | 1021.99 ± 4.00 |  |  | 1021.38 |  |  |
| Wind velocity (m/s) | 2.64 ± 1.12 | 0.90 | 1.88 | 2.33 | 3.01 | 8.66 |
| Q1 | 1.63 ± 0.19 |  |  | 1.66 |  |  |
| Q2 | 2.11 ± 0.13 |  |  | 2.11 |  |  |
| Q3 | 2.63 ± 0.20 |  |  | 2.61 |  |  |
| Q4 | 4.24 ± 1.05 |  |  | 3.93 |  |  |
| Relative humidity (%) | 65.70 ± 11.17 | 30.70 | 57.55 | 65.42 | 74.12 | 98.11 |
| Q1 | 51.37 ± 4.78 |  |  | 52.36 |  |  |
| Q2 | 61.76 ± 2.23 |  |  | 61.91 |  |  |
| Q3 | 69.51 ± 2.48 |  |  | 69.46 |  |  |
| Q4 | 80.19 ± 4.29 |  |  | 79.55 |  |  |
| Sunshine duration (h) | 6.33 ± 1.50 | 1.13 | 5.24 | 6.41 | 7.37 | 10.94 |
| Q1 | 4.39 ± 0.69 |  |  | 4.53 |  |  |
| Q2 | 5.88 ± 0.35 |  |  | 5.91 |  |  |
| Q3 | 6.87 ± 0.27 |  |  | 6.85 |  |  |
| Q4 | 8.21 ± 0.69 |  |  | 8.03 |  |  |
| Temperature (℃) | 13.32 ± 9.89 | -9.27 | 3.56 | 14.92 | 22.27 | 28.92 |
| Q1 | 0.06 ± 2.05 |  |  | 0.42 |  |  |
| Q2 | 9.08 ± 3.26 |  |  | 8.35 |  |  |
| Q3 | 18.81 ± 2.42 |  |  | 19.47 |  |  |
| Q4 | 25.31 ± 1.62 |  |  | 25.36 |  |  |
| Diurnal temperature variation (℃) | 9.12 ± 1.98 | 3.20 | 7.84 | 9.15 | 10.60 | 14.08 |
| Q1 | 6.51 ± 1.09 |  |  | 6.87 |  |  |
| Q2 | 8.47 ± 0.37 |  |  | 8.45 |  |  |
| Q3 | 9.87 ± 0.43 |  |  | 9.88 |  |  |
| Q4 | 11.55 ± 0.69 |  |  | 11.44 |  |  |

*M*: mean; Q: quartile; *SD*: standard deviation; *Min*: minimum; *Max*: maximum.

Table S2. Spearman correlation coefficient (*r_s_*) between monthly classified meteorological variables in Shandong Province, 2009–2017

|  | Precipitation | Atmospheric pressure | Wind velocity | Relative humidity | Sunshine duration | Temperature | Diurnal temperature variation |
| --- | --- | --- | --- | --- | --- | --- | --- |
| Precipitation | 1.00 |  |  |  |  |  |  |
| Atmospheric pressure | -0.41** | 1.00 |  |  |  |  |  |
| Wind velocity | -0.08** | -0.14** | 1.00 |  |  |  |  |
| Relative humidity | 0.57** | -0.11** | -0.26** | 1.00 |  |  |  |
| Sunshine duration | 0.00 | -0.19** | 0.26** | -0.37** | 1.00 |  |  |
| Temperature | 0.69** | -0.45** | -0.17** | 0.45** | 0.31** | 1.00 |  |
| Diurnal temperature variation | -0.29** | 0.02* | -0.19** | -0.57** | 0.49** | 0.00 | 1.00 |

** *P*<0.01, * *P*<0.05

Table S3. Relative risk (RR, 95%CI) of meteorological factors in total model and six sub-models in Shandong Province, 2009–2017.

| Variables | Total | Gender (male) | Gender (female) | Age (<5) | Age (20–39) | Occupation (scattered children) | Occupation (farmer) |
| --- | --- | --- | --- | --- | --- | --- | --- |
| Precipitation Q2 | 1.39(1.23,1.57) | 1.39(1.22,1.58) | 1.36(1.18,1.57) | 1.35(1.17,1.56) | 1.48(1.28,1.72) | 1.38(1.19,1.59) | 1.47(1.24,1.74) |
| Precipitation Q3 | 1.19(1.03,1.39) | 1.23(1.05,1.44) | 1.11(0.93,1.33) | 1.12(0.95,1.33) | 1.24(1.03,1.49) | 1.13(0.95,1.35) | 1.31(1.06,1.61) |
| Precipitation Q4 | **1.57(1.29,1.91)** | 1.63(1.32,2.01) | 1.47(1.17,1.86) | 1.57(1.25,1.97) | 1.58(1.24,2.01) | 1.57(1.24,1.97) | 2.00(1.52,2.64) |
| Atmospheric pressure Q2 | 1.00(0.82,1.22) | 1.08(0.87,1.33) | 1.05(0.85,1.32) | 1.08(0.87,1.34) | 1.05(0.82,1.35) | 1.06(0.85,1.32) | 1.14(0.87,1.48) |
| Atmospheric pressure Q3 | 0.83(0.65,1.07) | 0.87(0.67,1.14) | 0.95(0.72,1.26) | 0.98(0.74,1.29) | 0.81(0.59,1.12) | 0.93(0.70,1.23) | 1.00(0.71,1.40) |
| Atmospheric pressure Q4 | **0.54(0.39,0.73)** | 0.56(0.41,0.78) | 0.66(0.47,0.94) | 0.67(0.48,0.95) | 0.55(0.37,0.81) | 0.63(0.44,0.89) | 0.75(0.49,1.14) |
| Wind velocity Q2 | 1.43(1.25,1.64) | 1.36(1.17,1.58) | 1.44(1.22,1.70) | 1.37(1.18,1.61) | 1.48(1.23,1.79) | 1.34(1.15,1.57) | 1.51(1.23,1.86) |
| Wind velocity Q3 | 1.85(1.57,2.18) | 1.74(1.46,2.08) | 1.91(1.57,2.32) | 1.80(1.49,2.17) | 1.91(1.54,2.37) | 1.77(1.47,2.14) | 2.04(1.61,2.59) |
| Wind velocity Q4 | **2.00(1.59,2.52)** | 1.95(1.53,2.49) | 2.00(1.54,2.61) | 1.79(1.38,2.32) | 2.38(1.79,3.18) | 1.72(1.32,2.24) | 2.34(1.70,3.22) |
| Relative humidity Q2 | 0.76(0.66,0.88) | 0.77(0.67,0.90) | 0.78(0.67,0.93) | 0.77(0.66,0.91) | 0.78(0.65,0.92) | 0.77(0.65,0.90) | 0.76(0.63,0.93) |
| Relative humidity Q3 | 0.56(0.47,0.67) | 0.57(0.47,0.69) | 0.60(0.49,0.74) | 0.54(0.44,0.67) | 0.58(0.46,0.72) | 0.53(0.43,0.65) | 0.56(0.44,0.72) |
| Relative humidity Q4 | **0.49(0.38,0.63)** | 0.50(0.38,0.66) | 0.51(0.38,0.69) | 0.47(0.35,0.63) | 0.43(0.31,0.60) | 0.46(0.34,0.61) | 0.39(0.27,0.56) |
| Sunshine duration Q2 | 1.03(0.89,1.20) | 0.96(0.81,1.13) | 1.19(0.99,1.42) | 0.90(0.76,1.07) | 1.16(0.95,1.41) | 0.90(0.76,1.07) | 1.14(0.90,1.43) |
| Sunshine duration Q3 | 1.46(1.24,1.73) | 1.37(1.14,1.65) | 1.74(1.42,2.13) | 1.22(1.00,1.48) | 1.90(1.52,2.36) | 1.19(0.98,1.45) | 1.71(1.34,2.19) |
| Sunshine duration Q4 | **2.23(1.82,2.73)** | 2.07(1.66,2.58) | 2.56(2.01,3.26) | 1.58(1.25,2.01) | 2.78(2.14,3.62) | 1.61(1.27,2.05) | 2.41(1.79,3.25) |
| Temperature Q2 | 0.79(0.69,0.91) | 0.80(0.69,0.93) | 0.78(0.66,0.92) | 0.72(0.61,0.84) | 0.86(0.72,1.02) | 0.72(0.61,0.85) | 0.92(0.75,1.12) |
| Temperature Q3 | 0.54(0.44,0.65) | 0.51(0.42,0.63) | 0.58(0.46,0.72) | 0.56(0.45,0.70) | 0.58(0.46,0.73) | 0.55(0.44,0.68) | 0.64(0.49,0.83) |
| Temperature Q4 | **0.48(0.38,0.61)** | 0.44(0.34,0.56) | 0.52(0.39,0.69) | 0.57(0.44,0.76) | 0.43(0.32,0.59) | 0.57(0.43,0.75) | 0.46(0.33,0.66) |
| Diurnal temperature variation Q2 | 1.45(1.24,1.70) | 1.50(1.26,1.78) | 1.48(1.21,1.79) | 1.49(1.24,1.80) | 1.37(1.11,1.70) | 1.49(1.23,1.80) | 1.57(1.23,2.02) |
| Diurnal temperature variation Q3 | 1.53(1.25,1.86) | 1.52(1.22,1.89) | 1.53(1.20,1.95) | 1.63(1.29,2.06) | 1.30(0.99,1.69) | 1.61(1.27,2.04) | 1.39(1.02,1.88) |
| Diurnal temperature variation Q4 | **1.66(1.30,2.13)** | 1.67(1.28,2.19) | 1.72(1.28,2.32) | 1.77(1.33,2.35) | 1.44(1.04,2.00) | 1.74(1.30,2.33) | 1.68(1.16,2.44) |

Q: quartile. The relative risk (RR) of meteorological factors takes the Lowest value of variable (Q1) as a reference.

Table S4. Estimated hyperparameters, and measures of model fit in Shandong Province, 2009–2017.

|  | Total | Gender (male) | Gender (female) | Age (<5) | Age (20–39) | Occupation (scattered children) | Occupation (farmer) |
| --- | --- | --- | --- | --- | --- | --- | --- |
| **Hyperparameter** |  |  |  |  |  |  |  |
| Size for nbinomial zero-inflated observations | 0.308 | 0.320 | 0.290 | 0.295 | 0.311 | 0.290 | 0.260 |
| Zero-probability parameter for zero-inflated nbinomial | 0.004 | 0.006 | 0.009 | 0.008 | 0.010 | 0.01 | 0.018 |
| Precision for BYM model (iid spatial) | 1820 | 2000 | 2020 | 1960 | 2140 | 2050 | 2080 |
| Precision for BYM model (iCAR spatial) | 0.710 | 0.752 | 0.483 | 0.691 | 0.515 | 0.680 | 0.678 |
| Precision for rw2 (year) | 18400 | 19300 | 17000 | 17400 | 19800 | 15800 | 20000 |
| Precision for IID model (year) | 0.379 | 0.401 | 0.318 | 0.419 | 0.310 | 0.395 | 0.276 |
| **measure of model fit** |  |  |  |  |  |  |  |
| DIC | 28176.62 | 21827.23 | 18290.73 | 19461.26 | 15044.45 | 19170.92 | 12533.60 |
| p.eff (DIC) | 141.41 | 136.86 | 128.96 | 133.94 | 136.78 | 132.32 | 124.93 |
| WAIC | 28228.73 | 21866.92 | 18324.74 | 19494.01 | 15077.62 | 19202.19 | 12552.00 |
| p.eff (WAIC) | 178.01 | 162.91 | 150.33 | 153.18 | 156.59 | 150.41 | 132.31 |
| minus the sum of the log-values of CPO | 14119.46 | 10937.48 | 9165.135 | 9750.549 | 7541.64 | 9604.54 | 6278.54 |
| MLIK | -14306.09 | -11118.35 | -9342.76 | -9927.56 | -7723.56 | -9783.25 | -6443.68 |
| MSE | 16.38 | 5.67 | 3.29 | 4.89 | 2.07 | 4.84 | 1.46 |

BYM: Besag–York–Molliè; IID: independent and identically distributed; iCAR: intrinsic conditional autoregressive; rw2: random walk of order 2; DIC: deviance information criterion; p.eff: Effective number of parameters; WAIC: Watanabe–Akaike information criterion; CPO: conditional predictive ordinate; MLIK: marginal log-likelihood; MSE: mean square error.

Table S5. The RR and posterior probability values of medium-high-risk counties (districts) in Shandong Province, 2009–2017.

| County ID | County (District) | City | RR | Probability |
| --- | --- | --- | --- | --- |
| 1 | Lixia | Jinan | 2.848 | 0.925 |
| 2 | Shizhong | Jinan | 2.347 | 0.735 |
| 3 | Huaiyin | Jinan | 3.465 | 0.995 |
| 4 | Tianqiao | Jinan | 3.300 | 0.994 |
| 5 | Licheng | Jinan | 2.477 | 0.823 |
| 9 | Shanghe | Jinan | 2.837 | 0.941 |
| 12 | Huangdao | Qingdao | 2.351 | 0.772 |
| 28 | Shizhong | Zaozhuang | 2.964 | 0.960 |
| 30 | Yicheng | Zaozhuang | 2.860 | 0.928 |
| 31 | Taierzhuang | Zaozhuang | 5.551 | 1.000 |
| 62 | Rencheng | Jining | 2.656 | 0.930 |
| 63 | Weishan | Jining | 2.588 | 0.874 |
| 64 | Yutai | Jining | 2.939 | 0.950 |
| 89 | Lanshan | Linyi | 2.538 | 0.876 |
| 90 | Luozhuang | Linyi | 2.867 | 0.942 |
| 95 | Cangshan | Linyi | 2.620 | 0.903 |
| 112 | Dongchangfu | Liaocheng | 4.040 | 1.000 |
| 117 | Guan Xian | Liaocheng | 2.485 | 0.816 |
| 127 | Mudan | Heze | 2.315 | 0.739 |
| 128 | Caoxian | Heze | 2.900 | 0.961 |
| 131 | Juye | Heze | 3.306 | 0.996 |
| 134 | Dingtao | Heze | 2.333 | 0.726 |


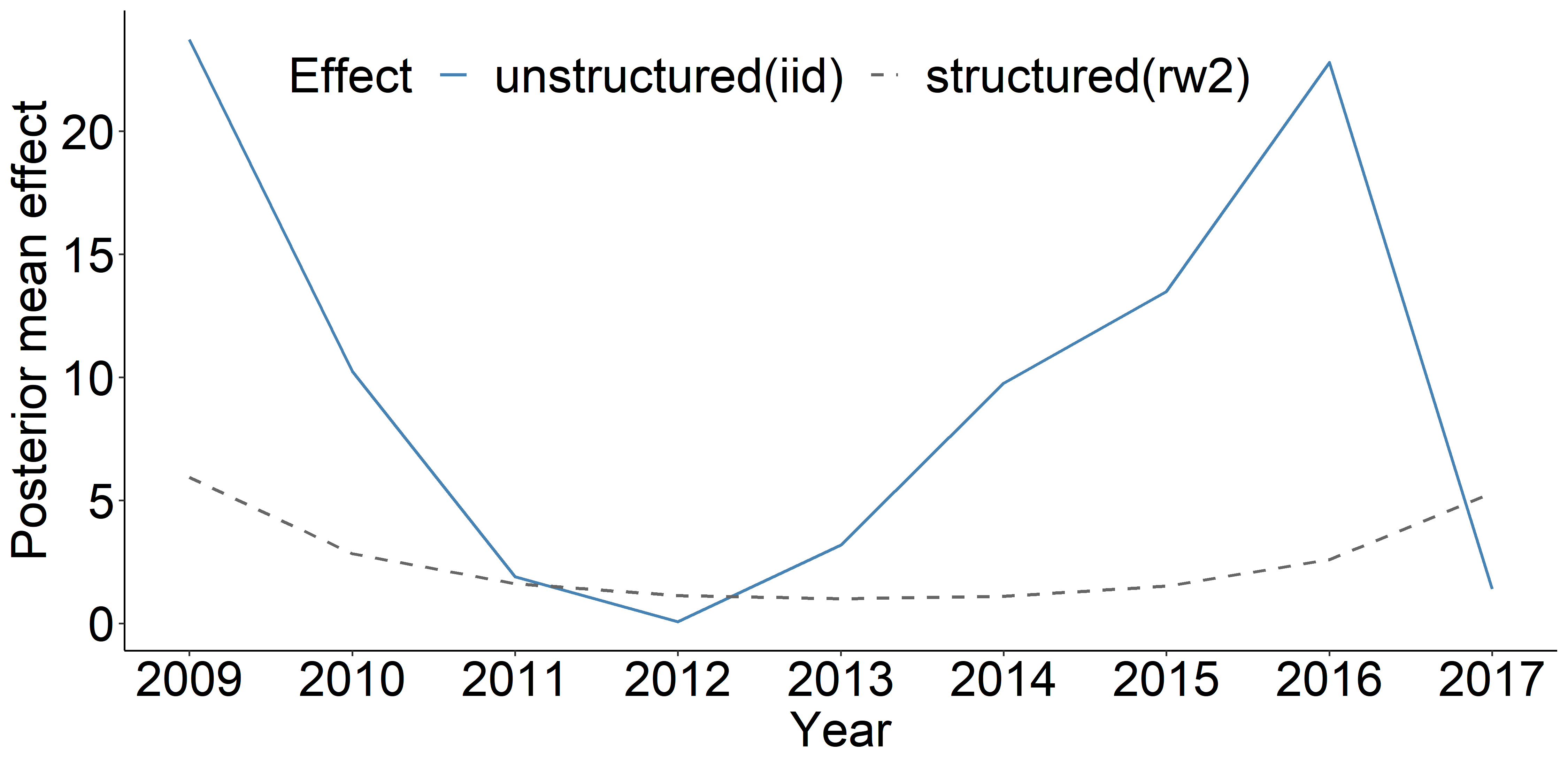


Figure S1. Posterior time trend for measles in Shandong Province, 2009–2017.


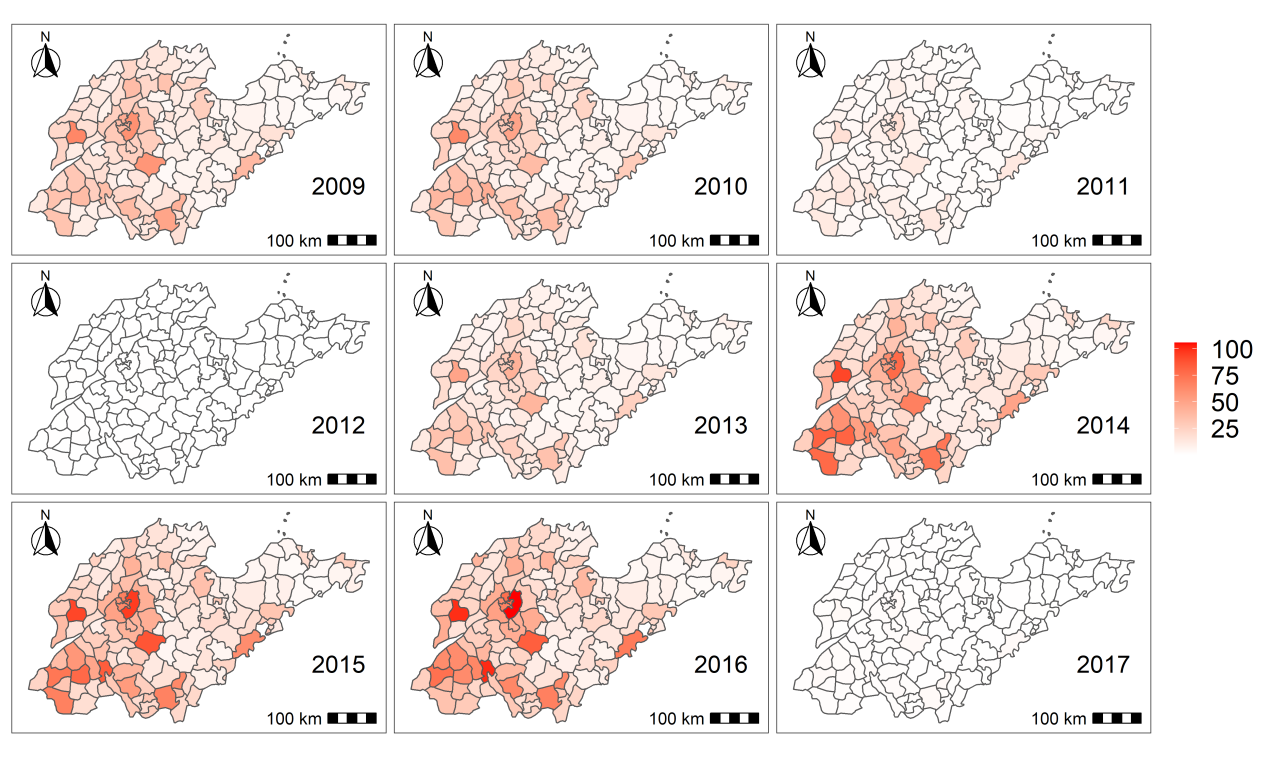


Figure S2. Model fitting value maps of measles cases in counties (districts) of Shandong Province, 2009–2017 (the gray area in maps is the Changdao county).
